# Organic Nitrates Favor Regression of Left Ventricular Hypertrophy in Hypertensive Patients on Chronic Peritoneal Dialysis

**DOI:** 10.3390/ijms14011069

**Published:** 2013-01-07

**Authors:** Han Li, Shixiang Wang

**Affiliations:** 1 Department of Blood Purification, Beijing Chao-Yang Hospital, Capital Medical University, Beijing 100020, China; E-Mail: lihandp@yahoo.com.cn; 2 Nephrology Faculty, Capital Medical University, No.8 Gongti South Road, Chaoyang District, Beijing 100020, China

**Keywords:** nitrate, ADMA, hypertension, left ventricular hypertrophy, renal dialysis

## Abstract

The aim of the study was to evaluate the effect of nitrates on left ventricular hypertrophy (LVH) in hypertensive patients on chronic peritoneal dialysis (PD). Sixty-four PD patients with hypertension were enrolled in this study. All patients accepted antihypertensive drugs at baseline. Thirty-two patients (nitrate group) took isosorbide mononitrate for 24 weeks. The remaining 32 patients (non-nitrate group) took other antihypertensive drugs. Blood pressure (BP), left ventricular mass index (LVMI) and plasma asymmetric dimethylarginine (ADMA) were monitored. Subjects with normal renal function were included as the control group (*n* = 30). At baseline, plasma ADMA levels in PD patients were significantly higher than the control group, but there was no significant difference in plasma ADMA levels between the two groups. At the end of the 24-week period, BP, LVMI, LVH prevalence and plasma ADMA levels in the nitrate group were significantly lower than those in the non-nitrate group. BP did not show a significant difference between 12 and 24 weeks in the nitrate group with a reduced need for other medication. Logistic regression analysis showed that nitrate supplementation and SBP reduction were independent risk factors of LVMI change in PD patients after adjusting for age, gender, diabetes history and CCB supplementation. It was concluded that organic nitrates favor regression of LVH in hypertensive patients on chronic peritoneal dialysis, and nitrates may be considered for use before employing the five other antihypertensive agents other than nitrates.

## 1. Introduction

Several studies have reported a high prevalence of cardiovascular disease in patients with end-stage renal disease (ESRD). The projected life expectancy of ESRD patients on dialysis is 20%~25% of that of the general population [1,2]. Furthermore, in ESRD patients, left ventricular hypertrophy (LVH) is considered the most important predictor of prognosis [3]. The high blood pressure (BP) frequently seen in ESRD patients is thought to be refractory in nature. Most ESRD patients with hypertension need to use three or more kinds of antihypertensive drugs to control the blood pressure. Although patients with chronic renal failure commonly have accompanying diseases which themselves have a high cardiovascular risk, such traditional risk factors account for only part of the very high cardiovascular morbidity and mortality in these patients. An expert panel from the USA National Kidney Foundation has recently identified the need for observational studies to ascertain the relation between established cardiovascular risk factors and cardiovascular outcomes and to identify new risk factors as a research priority.

Asymmetric dimethylarginine (ADMA), an endogenous inhibitor of nitric oxide (NO) production, is an important risk factor for cardiovascular disease and mortality in the ESRD population [1]. ADMA accumulation in the ESRD population is a consequence of reduced renal excretion and impaired enzymatic degradation and is related to the progression of atherosclerosis [2]. Nitrate can lead to vasodilatation by releasing NO, which is widely used in coronary artery disease. However, whether nitrate can control blood pressure (BP), reverse left ventricular hypertrophy (LVH), and decrease plasma ADMA in chronic continuous ambulatory peritoneal dialysis (CAPD) patients remains unclear. To investigate the effect of nitrate in CAPD patients, the presented prospective open-label, randomized controlled study was conducted.

## 2. Results and Discussion

### 2.1. Results

#### 2.1.1. Subject Characteristics

Table 1 summarizes the clinical characteristics of the study cohort. Sixty-four CAPD patients (28 male, 36 female) with a mean age of 56.2 ± 11.6 years (range 28–71 years) and a mean dialysis duration of 50.3 ± 27.2 months (range 5–98 months), were recruited. The aetiology for ESRD were chronic glomerulonephritis (28 cases), hypertensive renal disease (15 cases), diabetic renal disease (16 cases) and unidentified (5 cases). Patients were randomly grouped by computer-generated random numbers into the nitrate group (*n* = 32) or non-nitrate group (*n* = 32). There was no significant difference between the two groups in terms of age, sex ratio, dialysis duration, smoking, medication kinds of daily antihypertensive drugs, amount of daily antihypertensive drugs, KT/V, plasma levels of ADMA, Hb, creatinine, BUN, TG, TC, *etc.*, at baseline (*p* > 0.05).

#### 2.1.2. Influence of Nitrate on BP, Kinds and amount of Antihypertensive Drugs in CAPD Patients

Table 2 shows that BP levels decreased in both groups after 4, 8, 12 and 24 weeks of treatment. At the end of the 24-week period, the blood pressure (SBP, DBP and MAP), kinds and amount of antihypertensive drugs in the nitrate group were significantly lower than that in the non-nitrate group. After adjusting for diabetes, the blood pressure (SBP, DBP and MAP), kinds and amount of antihypertensive drugs in the nitrate group still remained lower than that in the non-nitrate group. However, there was no statistical significance in the response rate and control rate of BP in either group (response rate: 93.8% *vs.* 84.4%, χ^2^ = 1.444, *p* = 0.230; control rate: 65.6% *vs.* 50.0%, χ^2^ = 1.602, *p* = 0.206). SBP, DBP and MAP did not shown any significant difference between 12 and 24 weeks in the nitrate group.

The prescription of renin-angiotensin system (RAS)–blocking agents and CCB at baseline and throughout the study in the two groups is also listed in Table 2. There were no significant differences in the prescription of RAS-blocking agents at baseline and throughout the study in either group. Likewise, no significant differences were seen in the prescription of CCB at baseline, week 4, 8 and 12 of treatment in either group. However, after 24-weeks of treatment, the prescription of CCB in the nitrate group was significantly lower than that in the non-nitrate group.

#### 2.1.3. Influence of Nitrates on LVMI in CAPD Patients

Table 3 shows that after the 24 week of treatment, LVMI in both nitrate group and non-nitrate group were significantly decreased, from 67.5 ± 15.8 g/m^2.7^ to 46.2 ± 6.5 g/m^2.7^, and from 61.8 ± 12.5 g/m^2.7^ to 50.9 ± 8.0 g/m^2.7^, respectively. It was interesting to note that the LVMI in the nitrate group was significantly lower than that in the non-nitrate group. After adjusting for diabetes, the LVMIs in the nitrate group still remained lower than that in the non-nitrate group. The prevalence of LVH in the nitrate and non-nitrate groups decreased by 40.6% and 12.5%, respectively. There was a significant difference in both groups at 24 weeks (χ^2^ = 4.016, *p* = 0.045).

#### 2.1.4. Influence of Nitrates on Plasma ADMA Levels in CAPD Patients

Table 4 shows that the plasma ADMA level in CAPD patients was significantly higher than the control group at baseline, but there was no significant difference in plasma ADMA level between the nitrate group and non-nitrate group. At 24 weeks, the plasma ADMA level in the nitrate group was significantly lower than that in non-nitrate group. After adjusting for diabetes, the ADMA level in the nitrate group still remained lower than that in the non-nitrate group.

Partial correlation analysis showed that LVMI change was negatively correlated with plasma ADMA level (*r* = −0.307, *p* = 0.014). Logistic regression analysis showed that nitrate supplementation and SBP reduction were the independent risk factors of LVMI change in CAPD patients after adjusting for age, gender, diabetes history and CCB supplementation. See Table 5.

#### 2.1.5. Adverse Events in this Study

One CAPD patient suffered from headaches after nitrate administration, which was relieved after reduction. The incidence of adverse events was 3.1%.

### 2.2. Discussion

Hypertension is a complication commonly seen in patients with chronic kidney diseases. The incidence of hypertension grows along with the decrease in glomerular filtration rate (GFR). It was reported that the incidence of hypertension in patients with GFR less than 60 mL/min was 50%~75% [3]. However, the incidence of hypertension was extraordinarily higher in dialysis patients. In 69 dialysis units in the United States, almost 86% of the dialysis patients suffered from hypertension, and the control rate for their BP was merely 30% [3]. Hypertension is a significant risk factor for cardiovascular disease in CAPD patients. Foley *et al.* [4] found that with each 10 mmHg increase of BP in dialysis patients, the risk of LVH increased by 48%, ischemic heart disease increased by 39% and congestive cardiac failure increased by 44%. Cardiovascular disease was the primary cause of death in dialysis patients, and mortality from cardiovascular disease in dialysis patients was much higher than in the normal population [5,6].

ADMA is an endogenous inhibitor of nitric-oxide synthase [7]. Concentrations of ADMA are related to endothelial dysfunction in hypercholesterolaemic individuals [8]. ADMA is not excreted in patients with chronic renal failure, resulting in concentrations of these substances in plasma two to six times higher in uraemic patients than in healthy control individuals. Plasma level of ADMA is a strong and independent determinant of IMT of the carotid artery in the large number of subjects without overt cerebro-cardiovascular diseases [9]. Of note, ADMA concentrations are higher in dialysis patients who clinically manifest atherosclerosis than in those without atherosclerotic disease, which suggests that accumulation of ADMA might be an important cardiovascular risk factor in ESRD [10,11]. Many causes of hypertension in ESRD patients are, volume overload [12,13], activation of the RAS [14], sympathetic hyperactivity [15,16] and an increase in inhibitors of nitric oxide (NO) in the blood circulation, such as ADMA, which results in a high incidence of hypertension and difficulties in BP control [17–19]. CAPD patients with refractory hypertension need to be treated with combinations of three or more kinds of antihypertensive drugs [20]. Nitrates, as donors of NO, can dilate the smooth muscles of both veins and arteries, which can decrease the cardiac preload, and the left ventricular end-diastolic pressure to reduce the cardiac afterload. In the present study, it was interesting to note that nitrates were effective in CAPD patients with refractory hypertension who need combinational therapy of three or more kinds of antihypertensive drugs. Nitrates can increase the BP control rate, as well as reduce the total kinds and amount of antihypertensive drugs.

Under physiological conditions, the endothelium continuously generates NO, thus maintaining the circulatory system in a state of active vasodilatation. NO has a protective role for the cardiovascular system because it not only modulates arterial compliance and peripheral vascular resistance, but also inhibits vascular muscle cell proliferation, platelet aggregability and adhesion of monocytes to the endothelium—all processes that trigger atherosclerosis. When NO production is decreased, atherosclerosis ensues [21]. NO is generated from its precursor l-arginine via the enzyme activity of nitric oxide synthase. It was discovered that increased ADMA in the circulation of ESRD patients can competitively inhibit nitrogen monoxide production from l-arginine, which in turn increases vascular resistance and leads to endothelium dysfunction, hypertension and LVH [22]. Therefore, ADMA is not only a uremic toxin, but also a strong factor causing endothelial dysfunction and atherosclerosis and a strong predictor of mortality.

As a donor of NO, nitrate can release NO with catalysis of glutathione transferase in smooth muscle cells. As the plasma NO concentration increases, endothelium dysfunction improves, along with the inhibition of smooth muscle cell proliferation and myocardial hypertrophy. It was confirmed in animal models that the nitrate might suppress LVH remodeling, and that the efficacy of long-term treatment was better than that of short-term therapy [23]. There have been no clinical studies of the effect of nitrates on LVH remodeling in CAPD patients. Thus our study showed for the first time that the LVMI of CAPD patients decreased from 61.8 ± 12.5 g/m^2.7^ to 50.9 ± 8.0 g/m^2.7^ after treatment with nitrates for 24 weeks. Logistic regression analysis showed that nitrate supplementation and SBP reduction were the independent risk factors of LVMI change in CAPD patients. Therefore, we argue that nitrates may contribute to the reversion of LVH, which is not dependant on the decrease of BP.

Furthemore, we observed a significant decrease in ADMA concentration in CAPD patients with nitrate treatment. However, its mechanism is not yet clear. Esposito C *et al.* [24] confirmed that higher levels of ADMA, modulated by other molecules, such as calcineurin inhibitor, may even affect kidney transplant outcome. ADMA significantly increased at six months post-transplantation on cyclosporine regimen, but was significantly lower among patients on sirolimus or everolimus. Sverdlov AL *et al.* [7] showed that ADMA predicted LVH independent of afterload in a normal aging population. In our present study, partial correlation analysis showed that LVMI change was negatively correlated with plasma ADMA level (*r* = −0.307, *p* = 0.014). However, logistic regression analysis did not show that ADMA was the independent risk factor of LVMI change in CAPD patients. On the other hand, ADMA levels in serum samples obtained in the course of dialysis was complicated. The red blood cells had been shown to contain large amounts of ADMA as demonstrated by Billecke *et al.* [25]. So it was concluded that adding organic nitrate to the antihypertensive regimen of CAPD patients, benefited them, in terms of blood pressure control, regression of left ventricular hypertrophy and plasma ADMA level reduction.

## 3. Experimental Section

### 3.1. Patients

Sixty-four ESRD patients (36 females and 28 males) on CAPD for at least three months, with age ≥18 years and BP higher than 140/90 mm Hg (1 mmHg = 0.133 kPa) after treatment with at least three kinds of antihypertensive drugs in sufficient dosage (maximum dose in the dispensatory) for two weeks were enrolled in this open-label, randomized controlled study. All the patients were clinically stable and free from rheumatic heart disease, congenital cardiopathy, complications with chronic infections and severe liver injury with alanine aminotransferase (ALT) and/or aspartate aminotransferase (AST) two times higher than the upper limits of normal. Participants were excluded if their BP increased to the level of malignant hypertension, or had cardiac or cerebral events during the observation period, or were unable to follow the trial protocol because of severe adverse effects. The patients performed four 2-liter exchanges a day using the Baxter TwinBag system. Dwell times were generally 4–6 h during the day and 8 h overnight.

Patients were recruited in the Department of Blood Purification of Beijing Chao-Yang Hospital, Capital Medical University in China. The patients were treated with regular antihypertensive drugs, including angiotensin-converting enzyme inhibitor (ACEI) or angiotensin receptor blocker (ARB), calcium channel blocker (CCB) and β-receptor blocker or α-receptor blocker.

As a normal control group, age and gender matched, 30 healthy individual (15 females and 15 males) were enrolled for measuring plasma ADMA in this study.

The study was approved by the ethics committee of Beijing Chaoyang Hospital, Capital Medical University, and written informed consent was obtained from each participant.

### 3.2. Groups

Participants were randomly assigned by computer-generated random number list to receive either nitrate (nitrate group, *n* = 32) or non-nitrate medication (non-nitrate group, *n* = 32) on the basis of primary antihypertensive drugs (including ACEI, ARB, CCB, β-receptor blocker or α-receptor blocker).

### 3.3. Treatments

The nitrate group was treated with sustained-release isosorbide mononitrate (Imdur; AstraZeneca [Wu Xi] Trading Co. Ltd., Wu Xi, China). The initial dosage was 15 mg daily and the dosage was adjusted once a week to the maximal dosage of 60 mg daily based on BP. The targets for systolic BP (SBP) and diastolic BP (DBP) were 110–139 mm Hg and 70–89 mm Hg (1 mmHg = 0.133 kPa), respectively. The dosage of sustained-release isosorbide mononitrate was reduced or the participants removed in the following circumstances: (a) SBP remained below 110 mm Hg when other kinds of antihypertensive drugs (except ACEI and ARB) were withdrawn; (b) there was a severe adverse effect, which was hard to tolerate, such as headache. The entire duration of treatment was 24 weeks. During the observation period, all patients received other conventional therapy, including correction of anemia (target hemoglobin [Hb] ≥ 110g/L) and maintaining calcium and phosphorus balance.

### 3.4. Judgement of Medication Frequency

The medication amount was calculated by the defined daily dose (DDD). DDD was the mean daily dose needed in adults to achieve the main goals of treatment [26]. The medication amount of the antihypertensive drug was defined as the ratio of the daily consumption of a certain drug and the DDD value of the drug. The first choice of antihypertensive drug before recruitment was ACEI and ARB, the second was CCB, β-receptor blocker or α-receptor blocker.

### 3.5. Blood Pressure Measurements

Blood pressures (SBP and DBP) were measured in the morning, before the second daily fluid drainage of the abdominal cavity, by nursing staff using standard mercury sphygmomanometers on the right arm of seated participants who had rested for at least 5 min; three measurements 2 min apart were averaged.

### 3.6. Laboratory Investigations

To simulate the actual dialysis condition, all PD patients had a full abdomen at the time of blood sampling. Blood samples for laboratory measurements were drawn from the antecubital vein after the first 2 h of PD exchange with 1.5% dextrose dialysate in an overnight fasting state. Serum was separated immediately by lowspeed centrifugation (4000 rpm for 10 min at 4 °C) and used freshly for analysis by biochemists, who were blinded to classification of subjects as CAPD patients and controls.

Blood cell count, liver and renal function, blood glucose, blood lipid and electrolytes were measured by standard methods in the clinical laboratory.

### 3.7. ADMA Aassay

ADMA levels were measured using an ELISA kit (Protocol No: 07001, Cardiovasics, Palo Alto, CA, USA). Using a standard curve, the absorbance of the ADMA-antibody horse radish peroxidase complex in the sample was measured at 450 nm. ADMA concentrations of serum samples were determined in μmol/L.

### 3.8. Left Ventricular Mass Index (LVMI)

Echocardiography was performed to evaluate left ventricular mass index (LVMI) at baseline and after 4, 8, 12 and 24 weeks of treatment. Left ventricular end diastolic dimension (LVDD), interventricular septum thickness (IVST) and left ventricular posterior wall thickness (LVPWT) were measured. LVMI was calculated and normalized by height^2.7^ (LVMI = LVM/height^2.7^).

### 3.9. Criterion of Therapeutic Effect on BP and LVH

Full treatment response was defined as BP reaching below 140/90 mmHg, or mean arterial pressure decreasing by ≥15 mm Hg, or mean DBP decreasing by ≥20 mmHg. Partial response was defined as BP not falling below 140/90 mmHg, but the mean arterial pressure decreasing by ≥10 mmHg, or mean DBP decreasing by 10~19 mm Hg, or SBP alone decreasing by ≥30 mmHg. A nonresponse was defined as BP not reaching the above definition or in fact rising. Total response rate was defined as [(full responses + partial responses)/total cases] × 100%.

LVH was defined as LVMI >47 g/m^2.7^ in female or >50 g/m^2.7^ in male patients [27].

### 3.10. Data Analysis and Statistics

The SPSS version 13.0 statistics package was employed for the statistical analysis. Measurement data were presented as mean value ± standard deviation (±SD). Comparisons were performed using one-way ANOVA with *post hoc* analysis (LSD), independent-samples *t*-test or chi-square test. In addition, covariance analysis, partial correlation analysis and binary logistic regression analysis were performed. A *p* value < 0.05 was regarded as statistically significant.

## 4. Conclusions

Oral nitrate can effectively control BP, improve left ventricular hypertrophy and decrease plasma ADMA with safety and good tolerance in CAPD patients. Additionally nitrates may be considered for use before employing the five other antihypertensive agents other than nitrates.

## Figures and Tables

**Table 1:** Characteristics of both study groups.

Items	Nitrate group (*n* = 32)	Non-nitrate group (*n* = 32)	t/χ^2^ value	*p* value
Age, years	55.5 ± 11.2	56.8 ± 12.0	0.473	0.638
Sex, male/female	15/17	13/19	0.254	0.614
Dialysis duration, months	49.8 ± 28.3	50.8 ± 26.4	0.142	0.888
Smoking, no.(%)	3(9.4)	2(6.3)	0.217	0.641
Primary disease for ESRD				
Diabetes mellitus, no.(%)	5(15.6)	11(34.4)	3.000	0.083
Chronic glomerulonephritis, no.(%)	15(46.9)	13(40.6)	0.254	0.614
Hypertensive renal disease, no.(%)	8(25.0)	7(21.9)	0.087	0.768
Unidentified, no.(%)	1(3.1)	4(12.5)	1.854	0.173
SBP, mmHg	181.2 ± 16.0	181.9 ± 11.4	0.225	0.822
DBP, mmHg	99.5 ± 7.0	99.3 ± 5.6	0.098	0.922
MAP, mmHg	126.7 ± 9.4	126.9 ± 7.0	0.078	0.938
Kinds of daily antihypertensive drug	3.9 ± 0.6	3.8 ± 0.4	0.266	0.791
Amount of daily antihypertensive drugs	7.8 ± 1.1	7.7 ± 0.74	0.266	0.791
KT/V	2.3 ± 0.3	2.4 ± 0.4	0.134	0.894
LVMI, g/m^2.7^	67.5 ± 15.8	61.8 ± 12.5	1.623	0.110
LVH, no.(%)	24(75.0)	23(71.9)	0.080	0.777
ADMA, umol/L	0.91 ± 0.08	0.89 ± 0.08	0.764	0.448
Hb, g/L	115.4 ± 8.0	118.9 ± 8.8	1.647	0.105
Alb, g/L	32.5 ± 3.8	34.0 ± 4.1	1.456	0.150
Creatinine, umol/L	928.7 ± 246.2	960.6 ± 277.4	0.487	0.628
BUN, mmol/L	23.8 ± 6.3	22.2 ± 5.0	1.098	0.276
HsCRP, mmol/L	2.0 ± 1.0	1.6 ± 1.1	1.461	0.149
ALT, U/L	17.9 ± 6.7	17.0 ± 7.6	0.524	0.602
AST, U/L	19.2 ± 8.1	16.8 ± 7.5	1.211	0.230
TG, mmol/L	1.41 ± 0.60	1.39 ± 0.75	0.147	0.884
TC, mmol/L	4.0 ± 1.1	4.0 ± 0.8	0.219	0.827
LDL-C, mmol/L	2.2 ± 0.6	2.3 ± 0.6	0.431	0.668

Values are means ± SD, unless specified otherwise; SBP = systolic blood pressure; DBP = diastolic blood pressure; MAP = mean arterial pressure; LVMI = left ventricular mass index; LVH = left ventricular hypertrophy; Hb = hemoglobin; ADMA = asymmetric dimethylarginine.

**Table 2:** Comparison of BP before and after treatment in the two groups.

Items	Group	Case	Baseline	Week 4 of treatment	Week 8 of treatment	Week 12 of treatment	Week 24 of treatment	BP decrease after 24 weeks of treatment
SBP(mmHg)	nitrate	32	181.2 ± 16.0	166.7 ± 15.6 ab	147.5 ± 9.6 ab	143.3 ± 9.3 ab	140.2 ± 8.2 ab	41.0 ± 13.6
	non-nitrate	32	181.9 ± 11.4	170.7 ± 10.9 a	156.3 ± 15.8 a	149.2 ± 12.8 a	146.5 ± 12.1 a	35.5 ± 16.2

DBP(mmHg)	nitrate	32	99.5 ± 7.0	94.5 ± 6.9 ab	88.2 ± 6.6 ab	85.3 ± 6.9 ab	80. 9 ± 7.9 ab	18.5 ± 13.6 b
	non-nitrate	32	99.3 ± 5.6	95.3 ± 5.7 a	87.2 ± 6.5 a	86.3 ± 5.2 a	85.8 ± 5.8 a	13.6 ± 5.28

MAP(mmHg)	nitrate	32	126.7 ± 9.4	118.6 ± 9.2 ab	108.0 ± 6.9 ab	104. 6 ± 7.1 ab	100. 7 ± 7.2 ab	26.0 ± 6.9 b
	non-nitrate	32	126.9 ± 7.0	120.4 ± 7.0 ab	110.2 ± 8.6 ab	107.3 ± 6.7 ab	106. 0 ± 7.0 ab	20.9 ± 8.1

Kinds of daily antihypertensive drugs	nitrate	32	3.88 ± 0.55	4.88 ± 0.55 b	4.69 ± 0.47 a	3.72 ± 0.46 ab	3.63 ± 0.49 ab	
	non-nitrate	32	3.84 ± 0.37	4.84 ± 0.37 a	4.88 ± 0.34 a	4.25 ± 0.62 a	4.22 ± 0.66 a	

Amount of daily antihypertensive drugs	nitrate	32	7.74 ± 1.07	8.74 ± 1.07 a	9.74 ± 1.07 a	6.86 ± 1.12 ab	6.80 ± 1.15 ab	
	non-nitrate	32	7.56 ± 0.96	8.56 ± 0.96 a	9.56 ± 0.96 a	9.13 ± 0.84 a	9.14 ± 0.83 a	

RASI, no.(%)	nitrate	32	32 (100)	32 (100)	32 (100)	32 (100)	32 (100)	
	non-nitrate	32	32 (100)	32 (100)	32 (100)	32 (100)	32 (100)	

CCB, no.(%)	nitrate	32	32 (100)	32 (100)	32 (100)	24(75.0)	17(53.1) b	
	non-nitrate	32	32 (100)	32 (100)	32 (100)	29(90.6)	28(87.5)	

Values are means ± SD, or numbers (percentage); BP = blood pressure; SBP = systolic blood pressure; DBP = diastolic blood pressure; MAP = mean arterial pressure; CCB = calcium channel blocker; RASI = renin-angiotensin system inhibitor, including ACEI and ARB;

a *p* < 0.05, compared with baseline;

b *p* < 0.05, compared with non-nitrate group in corresponding period.

**Table 3:** Comparison of LVMI and LVH before and after treatment in the two groups.

Items	Group	Case	Baseline	Week 4 of treatment	Week 8 of treatment	Week 12 of treatment	Week 24 of treatment	LVMI decrease after 24 weeks of treatment
LVMI, g/m^2.7^	nitrate	32	67.5 ± 15.8	66.3 ± 15.3	63.4 ± 15.6	58.8 ± 15.7 a	46.2 ± 6.5 ab	14.6 ± 4.9
	non-nitrate	32	61.8 ± 12.5	60.5 ± 12.2	58.2 ± 12.4	53.5 ± 12.1 a	50.9 ± 8.0 a	10.6 ± 6.7

LVH, no.(%)	nitrate	32	24(75.0)				11(34.4)	
	non-nitrate	32	23(71.9)				19(59.4)	

Values are means ± SD, or numbers (percentage); LVMI = left ventricular mass index; LVH = left ventricular hypertrophy;

a *p* < 0.05, compared with baseline;

b *p* < 0.05, compared with non-nitrate group in corresponding period.

**Table 4:** ADMA Levels in CAPD Patients and controls.

Items	Nitrate group (*n* = 32)	Non-nitrate group (*n* = 32)	Control group (*n* = 30)
ADMA, umol/L			
At baseline	0.91 ± 0.08 a	0.89 ± 0.08 a	0.24 ± 0.04
24-weeks	0.66 ± 0.06 b	0.88 ± 0.08	

a *p* < 0.05, compared with control group;

b *p* < 0.05, compared with non-nitrate group.

**Table 5:** Logisitc regression analysis of risk factors of LVMI change in CAPD patients.

Items	Regression coefficient	Standard error	Wald χ^2^ value	*p* value	OR value	95% confidence limits	

						Lower limit	Upper limit
Nitrate Preparation	−1.532	0.609	6.322	0.012	0.216	0.066	0.713
SBP reduction	0.058	0.021	7.497	0.006	1.060	1.017	1.104

SBP = systolic blood pressure; OR = odds ratio.
